# Supplementary material for: The Pharmacokinetics in Mice and Cell Uptake of Thymus Immunosuppressive Pentapeptide Using LC-MS/MS Analysis
Source: Molecules. 2022 Jul 1;27(13):4256. doi: 10.3390/molecules27134256 (PMC9268305; doi:10.3390/molecules27134256)
Supplement: Supplementary file 1 [file molecules-27-04256-s001.zip › molecules-1784953-supplementary.pdf]

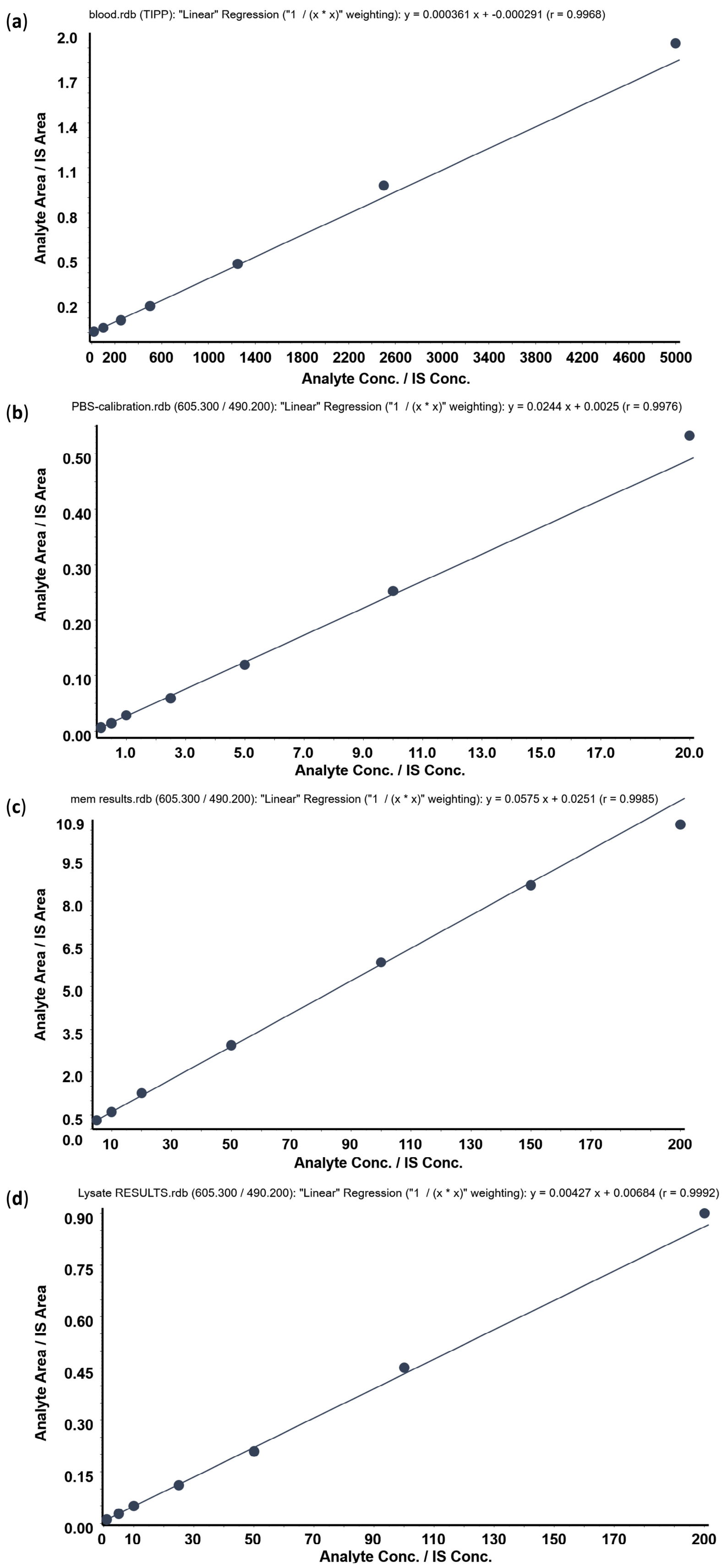

Figure S1. TIPP calibration curve and linearity. Linear calibration fit for TIPP in murine plasma (**a**), the final cell rinse (**b**), cell supernatants (**c**), and cell lysates (**d**).
